# Epitaxial deposition of silver ultra-fine nano-clusters on defect-free surfaces of HOPG-derived few-layer graphene in a UHV multi-chamber by *in situ *STM, *ex situ *XPS, and *ab initio *calculations

**DOI:** 10.1186/1556-276X-7-173

**Published:** 2012-03-06

**Authors:** Gebhu F Ndlovu, Wiets D Roos, Zhiming M Wang, Joseph KO Asante, Matete G Mashapa, Charl J Jafta, Bonex W Mwakikunga, Kenneth T Hillie

**Affiliations:** 1 National Centre for Nano-structured Materials, Council for Scientific and Industrial Research (CSIR), Meiring Naude Road, Brummeria, 395 Pretoria, 0001, South Africa; 2 Department of Physics, University of the Free State, 205 Nelson Mandela Drive, 339 Bloemfontein, 9300, South Africa; 3 State Key Laboratory of Electronic Thin Films and Integrated Devices, University of Electronic Science and Technology of China, Chengdu, 610054, People's Republic of China; 4 Department of Physics, Tshwane University of Technology, Private Bag X680 Pretoria, 0001, South Africa; 5 Department of Physics, University of Malawi-The Polytechnic, Private Bag 303, Chichiri, Blantyre, 0003, Malawi

## Abstract

The growth of three-dimensional ultra-fine spherical nano-particles of silver on few layers of graphene derived from highly oriented pyrolytic graphite in ultra-high vacuum were characterized using *in situ *scanning tunneling microscopy (STM) in conjunction with X-ray photoelectron spectroscopy. The energetics of the Ag clusters was determined by DFT simulations. The Ag clusters appeared spherical with size distribution averaging approximately 2 nm in diameter. STM revealed the preferred site for the position of the Ag atom in the C-benzene ring of graphene. Of the three sites, the C-C bridge, the C-hexagon hollow, and the direct top of the C atom, Ag prefers to stay on top of the C atom, contrary to expectation of the hexagon-close packing. *Ab initio *calculations confirm the lowest potential energy between Ag and the graphene structure to be at the exact site determined from STM imaging.

## Introduction

The control of material assembly on the nano-meter scale [1-4] as well as their self-assembly [5-7] are the two extremes of the most intensely studied subjects in the fields of nano-science and nano-technology. Self-assembly is characterized by high yields but often chaotic organization of nano-particles, whereas absolute control leads to accurate positioning, usually at the expense of yield. Controlled or tuneable self-assembly [8] balances the two extremes and is the most pursued subject for attainment of controlled properties and, at the same time, maintain mass production.

As the dimensions of metal particles decrease, novel properties different from those of the bulk counterparts are observed and ultimately resulted in the miniaturization of electronic devices [9]. Shrinking the size of devices drastically leads to the manifestation of unexpectedly enhanced electronic, optical, magnetic, chemical, and geometric properties [6,10,11].

On relatively inert substrates, such as graphite, many nano-structures can be fabricated in a nearly free-standing state by various deposition techniques such as thermal evaporation [12-14]. These structures can be characterized by popular analytical techniques such as scanning probe microscopy and X-ray photoelectron spectroscopy (XPS).

On a fundamental level, there are two recognized design strategies for the fabrication of supported nano-scale materials. These are classified as top-down approach, which entails various lithographic methods (working from the macroscopic to the nano-scopic), and bottom-up approach which involves the self-assembly of atoms or molecules on the surface into large structures [9,11]. This, for example, can be done by surface segregation. In general terms, bottom-up assembled nano-scale structures could provide unparalleled processing speed and size reductions, and hold the promise of powering future electronic devices. This approach totally opens up new opportunities beyond the limits of top-down technology through self assembly [9,15,16].

Highly oriented pyrolytic graphite (HOPG) has been extensively used as a prototypical inert and atomically flat model system in many nucleation and growth studies of silver (Ag) clusters and islands [11,17-20]. The use of HOPG as a substrate is advantageous in a sense that metallic properties of HOPG allow electron spectroscopy and microscopy experiments to be conducted without surface charging problems. For instance, atomic resolution scanning tunneling microscopy (STM) images can be easily obtained on HOPG.

Studies on properties of deposited silver nano-clusters and particles are not only of fundamental interest but also related to various technologically important fields such as catalysis, electronic devices, and gas sensors [21,22]. These metal clusters can be regarded as the precursors to a new generation of nano-structured materials and devices [10,13,23].

In the field of catalysis, ordered arrays of metal clusters show catalytic properties dependent upon cluster size and shape. The reactivity and catalytic activity can be very dependent upon geometry arrangement and electronic structure of the supported cluster [2,6]. STM is a very promising tool to study the supported metal clusters as it gives the complementary information about the particle/support morphology.

The nucleation and growth dynamics of Ag particles on an inert HOPG substrate is generally portrayed as in a three-dimensional (3-D) islanding mode (Volmer-Weber type of growth) which is often thermodynamically favored over uniform films or random-distributed adatoms as established by STM [23,24].

This article presents an investigation by STM and XPS of individual self-assembled Ag nano-clusters grown by thermal evaporation of Ag atoms on HOPG surfaces at room temperature. Density function theory (DFT) *ab initio *calculations are conducted to determine the site selective adsorption of Ag on the hexagonal graphene layer.

### Experimental details

Noting that the experimental variables that influence the dynamics of Ag nano-particles are temperature, deposition rate and the residual vacuum [20,25], the present experiments were carried out in two different ultra-high vacuum chambers. The first chamber was equipped with a Ag effusion cell, Omicron variable temperature (VT) STM (Omicron NanoTechnology GmbH, Taunusstein, Germany), low energy electron diffraction and Auger electron spectroscopy (AES) optics in UHV.

Before deposition, clean HOPG (0001) samples were prepared by cleaving in air and immediately transferring substrate into the VT-STM UHV chamber through a load lock. The cleanness of HOPG was confirmed by STM and AES prior to evaporation. Silver was evaporated by thermal evaporation (base pressure < 10^-10 ^Torr) of the solid material (99.9999% purity) from a Knudsen cell which gives a flux of atoms onto the HOPG substrate maintained at room temperature. The average deposition rate was 0.1 monolayer/sec and constitute a current flux of 1.04 μA that was allowed to flow for approximately 5 s. The flux of Ag atoms was kept constant by controlling the emission current between the W filament and the Ag source. Nano-meter-size Ag particles were spontaneously formed by diffusion and aggregation of the deposited material on the surface. AES confirmed the presence of Ag on the carbon dominated surface after growth. The samples were then studied by STM, operated in the constant current mode using electrochemically etched W tips as probes. Height, as a function of lateral position, represents the surface image. The STM-image scales were calibrated using Si(111)-7 × 7 and HOPG.

Electronic and chemical properties of deposited Ag clusters were studied using the second UHV chamber equipped with a XPS spectrometer. The Ag on HOPG samples was mounted in a PHI 5400 XPS vacuum chamber with a base pressure of < 8 × 10^-9 ^Torr. Angle-resolved XPS was performed using a non-monochromatic magnesium K_α _source (1,253.6 eV) and a concentric hemispherical sector analyzer with pass energy of 178.95 eV and a scan rate of 5 eVs^-1^. The X-ray source was operated at a power of 300 W at various electron take-off angles. The binding energy scale was calibrated with the Cu 3p_3/2 _(933 eV) and Au 4f_7/2 _(84 eV) peaks.

Within the framework of density function theory, density functional calculations were carried out using the generalized gradient approximations/PBE [26] exchange correlation potential embedded within the DMol^3 ^[27]. The interaction between the ionic cores and the valence electrons is modeled using the ultra-soft pseudopotentials of Vanderbilt [28].

Energetics and electronic properties, density of states and band structures were computed for the pristine (pure) graphene surface, silver adsorbed on the hollow site, bridge site of the hexagonal ring as well as on the top site of the carbon atom in the hexagon of the 33-atom graphene sheet. A kinetic energy cutoff of 350 eV was employed, and this choice gave fully converged calculations. Brillouin zone sampling was done using the 2 × 2 × 1 special k-point mesh employing the Monkhorst-Pack scheme [29].

## Results and discussion

The main experimental variables that influence the nano-Ag deposition dynamics are temperature, deposition rate, and the residual vacuum [20,25]. In order to achieve the goal of fabricating nano-structured materials that satisfy particular application requirements, it is essential to understand, at atomic scale, the basic thermodynamic and kinetic properties of deposited and nucleated species that determine the size, shape, and atomic structures of the self-assembled clusters [17,18]. To display the intrinsic properties of nano-structures, their interaction with the substrate should be significantly weaker than that in an epitaxial system and should be, however, strong enough to maintain mechanical bonding required for surface analysis. The sticking coefficient of silver on nearly perfect HOPG, even at low temperature, is reported to be far below unity [5,14]. Metal clusters have a stronger interaction among themselves compared to the weaker Ag-HOPG interaction [14,17,22]. These challenging factors have resulted on a rather rare studies of metal clusters deposited on nearly perfect HOPG substrates [8,17]. Previous XPS studies have shown that, as particle size becomes lower than about 5 to10 nm, shifts of metal core levels can often be observed [30-33]. The core level shifts may be influenced by the particle geometry since different geometries may change the number of atoms with different coordinations. This results in electronic structures different from the bulk [34-36].

### Morphology by scanning tunneling microscopy

**Figure 1:**
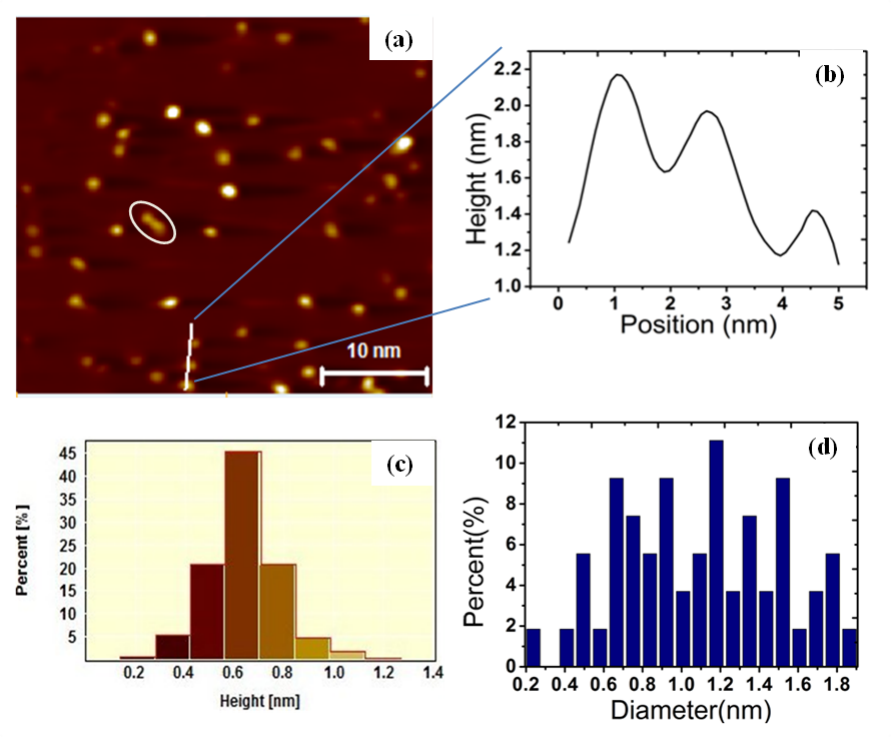
**STM images of Ag clusters**. (**a**) STM images (3 mV, 2 nA) of Ag clusters deposited onto a HOPG substrate illustrating the Volmer-Weber cluster growth, (**b**) line scan taken along the white line in (a). The graphs in (**c**) and (**d**) are histograms of particle height and particle diameter distributions, respectively.

Figure 1a shows an STM image of flat surface of HOPG with random distribution of isolated predominantly spherical particles of silver prepared by evaporating Ag at room temperature onto the HOPG substrate. All STM images were acquired in constant-current mode. The average particle size is about 1.2 nm in diameter and 0.68 nm in height (Figure 1c, d). The apparent particle height in STM images is a result of both electronic and geometric contrasts; thus, the obtained particle height using STM might contain some errors of a few nano-meters. A commercially available scanning probe image processor v5.110 software package from Image Metrology (Horsholm, Denmark) was used to measure the height and diameter distributions of the Ag nano-particles. The images were flattened before any size determinations to ensure that the influence of the substrate morphology is negligible. About 15% to 18% of the HOPG surface is estimated to be covered by Ag in the acquired STM data. This has been determined by counting the number of Ag particles in a unit area, which was then multiplied by the mean particle diameter, estimated by the widths of the half maxima of the particle profiles in STM images.

Even though the coverage determination is crude due to the in-homogeneity of the cluster spot, STM images show that Ag clusters exist as individual clusters on the surface. Previous studies have shown that diffusion and aggregation of clusters on the surface after deposition may change the cluster size distribution significantly such that the considerable effort of producing nano-size clusters may be compromised [37-39]. However, in this study, at coverages of approximately 0.5 ML of Ag on HOPG, there was no direct evidence from the STM images of cluster aggregation of the incident clusters at the surface. Similar experimental observations of Ag clusters on HOPG were made by Salido et al [40]. Due to the difference in lattice parameters between silver and HOPG substrate, the clusters experience a weak interaction with the substrate, implying that they remain sufficiently mobile and are able to diffuse within the HOPG surface. However, at a coverage of 0.5 ML of Ag, the movement of clusters is quite negligible [40-42]. Furthermore, the Ag clusters under consideration have average diameter of approximately 1.2 nm which means they are so closely analogous to a few atoms that cluster and contain delocalized conduction electrons which is a necessary requirement for quantum confinement [42]. Areas of bare HOPG between Ag clusters were evident from STM images. The particles grow three dimensionally via the Volmer-Weber growth mechanism. The white oblate shape superimposed on the STM image of Figure 1a shows two clusters merging through a nucleation process. The STM images show well-dispersed particle distributions on the surface, implying that these surfaces did not undergo particle agglomeration due to STM tip.

**Figure 2:**
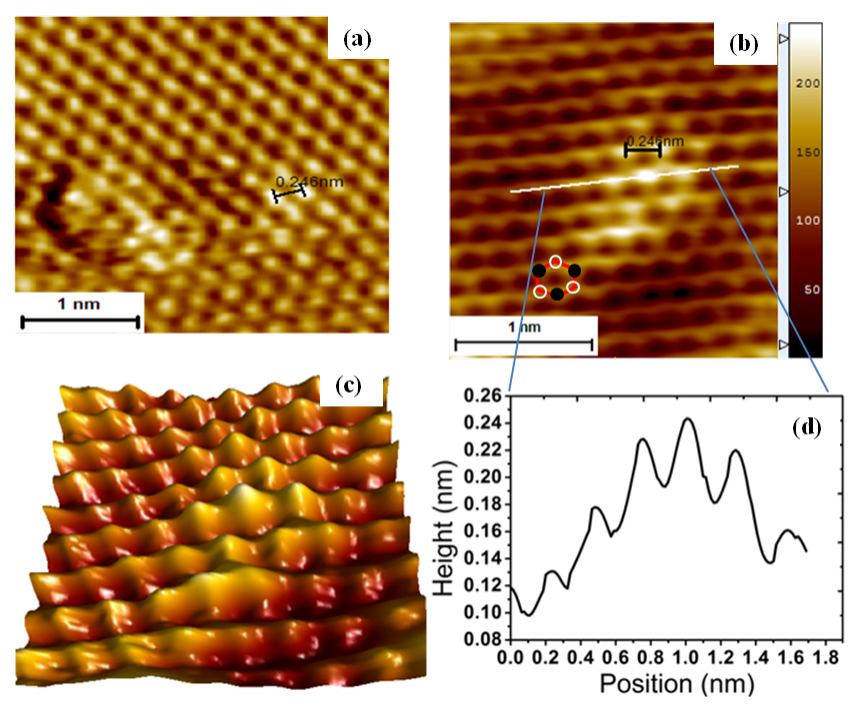
**STM image of Ag adatoms**. (**a**) High resolution STM image of Ag adatoms on HOPG (1.5 mV, 1.7 nA). (**b**) An image of a cluster of Ag adatoms showing the adsoption sites on HOPG. The superimposed hexagon illustrates the location of B-type (black circles) and A-type (open circle) carbon atom arrangement on HOPG. (**c**) 3-D view of the image in (b). A line scan (**d**) along the line in (b) shows the variation in height of the C atoms and Ag adatoms.

In order to illustrate the fact that the observed STM data of Ag clusters grown on HOPG were not due to tip artifacts, the sample surfaces were imaged in high resolution to obtain both Ag and HOPG atoms. Metal clusters belong to the mesoscopic system, systems composed of a countable number of atoms or molecules ranging from very few to a few thousand as illustrated by Figure 2a which is a 'zoom-in' image on an atomically resolved cluster of Ag atoms on the surface. The high-resolution STM image clearly shows the adsorption sites of Ag on the HOPG surface. The Ag adatoms appear to be sitting directly on the 'B' site of the HOPG as confirmed by DFT studies to follow. The line scan (Figure 2b) taken along the line in Figure 2a depicts the corrugations of both C and Ag atoms at the surface. The Ag-Ag measured distance is comparable to that of the spacing between C-C atoms on graphite (0.246 nm) as illustrated on the image. The previously anticipated agglomeration of clusters due to their mobility on inert substrate was not observed in this study.

### Local elemental analysis by X-ray photoelectron spectroscopy

**Figure 3:**
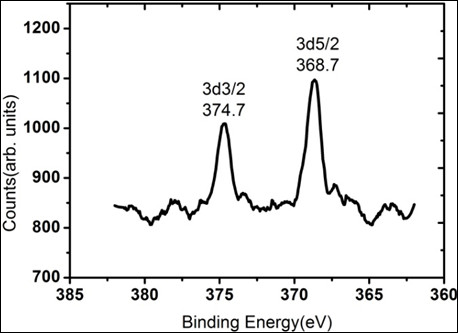
**Ag 3-D spectra of deposited Ag clusters on pristine HOPG prepared by thermal evaporation**.

High-energy resolution angle-resolved XPS spectra were collected as shown in Figure 3. No core level peaks were obtained at take-off angles above 10°, implying that the Ag was on the top most surface layer of HOPG. The 3-D core level peaks occur at 374.7 eV and 368.7 eV binding energies. The core level energy shift with respect to pure Ag amounts to about 0.5 eV to higher binding energy [43]. XPS studies on these Ag nano-particles reveal that the Ag 3-D binding energies are quite sensitive to the Ag particle size on graphite and show deviations from the bulk properties in terms of the Ag 3-D level binding energy.

One possible interpretation of the acquired STM data is that the Ag clusters exist as individual entities at lower Ag coverages. When particle size becomes smaller, the positive hole created by the photo-ionization process can be less efficiently screened, resulting in an additional positive core level shift (final state effect) which is evident from the observed shift in binding energy of the XPS data (Figure 3). Considering that the work function of HOPG (approximately 4.6 eV) is higher than those of Ag surfaces (approximately 4.2 eV) by about 0.4 eV, the Ag nano-particles can be partially positively charged on HOPG, resulting in the positive shifts [44,45]. There are other factors such as the induced lattice strain [46] which is a consequence of a decrease in particle size and/or, alternatively, the increased number of uncoordinated [32] atoms compared to the bulk, which might be used to explain the large positive core level shift of Ag on HOPG.

### *Ab initio *density function theory observations

**Figure 4:**
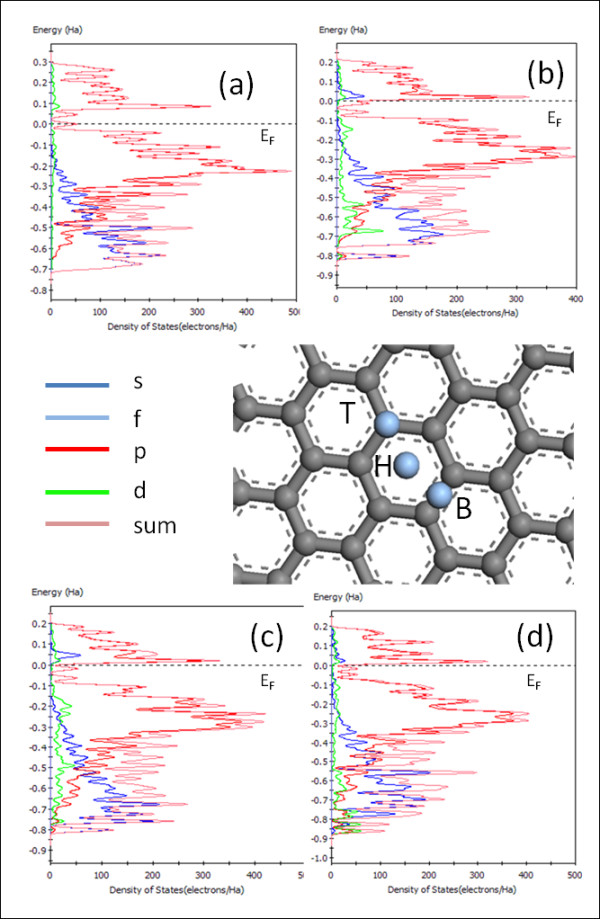
**Band gap characteristics**. Modeled 33-atom single layer of graphite with silver atoms adsorbed on different sites (center; T, ontop; H, hollow; and B, bridge). Band gap characteristics of (**a**) pristine graphene, (**b**) Ag atom on top sites, (**c**) Ag atom on hollow sites, and (**d**) Ag atom on bridge sites, respectively. Nothing significant for Ag on top of C, but the introduction of Ag on the bridge site (**c**) resulted in the disappearance of the silver signature around the Fermi level, and less significant peaks are observed very far from the Fermi level (around -0.55 eV) in the valence band region. The legends s, p, d, f and sum represents electronic energies from the s, p, d, and f orbitals, respectively, and sum represents the overall/total contribution, in other words, *E*_s _+ *E*_p _+ *E*_d _+ *E*_f _= *E*_sum_, where *E *represents the respective energies.

In order to confirm the preference of Ag sitting on top of C atoms rather than at the bridge or in the hollow sites (see Figure 4 center) of the C hexagon, an *ab initio *study was necessary. Upon full geometry optimization (structural relaxation) of the systems, silver atoms preferred to occupy on top sites (on top of the carbon atom). This was found to be the most energetically stable configuration (see Table 1) compared to the hollow and bridge sites.

**Table 1:** Corresponding calculated total energies of the system at different adsorption sites

Ag adsorption site	Potential energy (10^4 ^eV)
Relaxed graphene	-3.3161821
Ag atom on top of C atom in the hexagon ring, (labeled T in Figure 4)	-18.0004936
Ag atom on the hollow in the C-hexagon ring, (labeled as H in Figure 4)	-17.4627885
Ag atom on the C-C bridge in the C-hexagon ring (labeled as B in Figure 4)	-17.5661164

The effect of Ag on the electronic structure of graphene was further investigated (Figure 4a, b, c, d). Figure 4a shows the electronic density of states for the pristine graphene. The predicted direct band gap shows semi-metallic characteristic as can be seen from the band structure. Introducing silver on top sites induces acceptor levels at the bottom of the conduction band, and these states overlap across the Fermi, rendering the system metallic. This effect can be seen by the disappearance of the band gap between the valence and the conduction band as seen in Figure 4b. This study shows that the introduction of low concentration of metal clusters such as silver can help fine-tune the graphene band gap.

In Figure 4, the labels s, p, d, and f on the legend illustrate the electronic energies from s, p, d, and f orbitals which are defined by a common azimuthal quantum number *l *within an energy shell corresponding to *l *= 0, 1, 2, and 3, respectively. The sum depicts the overall energy contribution from orbital.

Considering the density of states, one observes that the contribution by the silver is encountered at the bottom of the conduction band for the silver on top of carbon (Figure 4b) and one with silver in the hollow site (Figure 4c). However, introducing silver on the bridge site (Figure 4c) resulted in the disappearance of the silver signature around the Fermi level, and less significant peaks are observed very far from the Fermi level (around -0.55 eV) in the valence band region.

## Conclusion

The ability of STM to access the morphology of small three-dimensional Ag clusters (*d *< 2 nm) supported on graphite was investigated. The structures were grown under well-controlled conditions in order to obtain clusters having well-defined morphology. The study suggests that the Ag clusters remain as individual entities after deposition with no sign of agglomeration. Using XPS, positive shifts of the Ag 3-D levels were observed. Ag/HOPG systems suggest that Ag particles are positively charged due to the charge transfers between substrates and metal nano-particles. A complete characterization of the morphology of the small clusters at the atomic level was possible. The study shows the preferred adsorption site (on top of C atoms) of Ag adatoms on graphene-terminated HOPG surface. The results consistently demonstrate that the Ag clusters do not disrupt atoms from the graphite surface layer. First principles of density functional theory were successfully employed to verify experimental observations. Adsorption of silver on the graphene sheet was investigated for different adsorption sites within the graphene sheets. It predicted that the most stable site was one where silver adsorbs on the top site of carbon, and this site was found to be energetically the most stable site.

## Competing interests

The authors declare that they have no competing interests.

## Authors' contributions

GFN, KTH and BWM designed and did all the deposition and STM experiments and *ab initio *calculations. WDR and CJJ performed the XPS analysis of the sample. MGM implemented the *ab initio *calculations. ZMW helped with useful discussion, and JKOA was also involved with XPS and deposition experiments and the writing of the manuscript. All authors read and approved the final manuscript.
